# Transcriptional profiling of *Saccharomyces cerevisiae* exposed to propolis

**DOI:** 10.1186/1472-6882-12-194

**Published:** 2012-10-24

**Authors:** Patrícia Alves de Castro, Marcela Savoldi, Diego Bonatto, Iran Malavazi, Maria Helena S Goldman, Andresa A Berretta, Gustavo Henrique Goldman

**Affiliations:** 1 Faculdade de Ciências Farmacêuticas de Ribeirão, Preto Universidade de São Paulo, São Paulo, Brazil; 2 Centro de Biotecnologia da UFRGS, Universidade Federal do Rio Grande do Sul, Porto Alegre, Brazil; 3 Departamento de Genética e Evolução, Centro de Ciências Biológicas e da Saúde (CCBS), Universidade Federal de São Carlos, Porto Alegre, Brazil; 4 Faculdade de Filosofia, Ciências e Letras de Ribeirão Preto, Universidade de São Paulo, São Paulo, Brazil; 5 Apis Flora Industrial e Comercial, Ribeirão Preto, São Paulo, Brazil; 6 Laboratório Nacional de Ciência e Tecnologia do Bioetanol – CTBE, Caixa Postal 6170, Campinas, São Paulo, 13083-970, Brazil

## Abstract

**Background:**

Propolis is a natural product of plant resins collected by honeybees (*Apis mellifera*) from various plant sources. Our previous studies indicated that propolis sensitivity is dependent on the mitochondrial function and that vacuolar acidification and autophagy are important for yeast cell death caused by propolis. Here, we extended our understanding of propolis-mediated cell death in the yeast *Saccharomyces cerevisiae* by applying systems biology tools to analyze the transcriptional profiling of cells exposed to propolis.

**Methods:**

We have used transcriptional profiling of *S. cerevisiae* exposed to propolis. We validated our findings by using real-time PCR of selected genes. Systems biology tools (physical protein-protein interaction [PPPI] network) were applied to analyse the propolis-induced transcriptional bevavior, aiming to identify which pathways are modulated by propolis in *S. cerevisiae* and potentially influencing cell death.

**Results:**

We were able to observe 1,339 genes modulated in at least one time point when compared to the reference time (propolis untreated samples) (*t-*test, *p-*value 0.01). Enrichment analysis performed by Gene Ontology (GO) Term finder tool showed enrichment for several biological categories among the genes up-regulated in the microarray hybridization such as transport and transmembrane transport and response to stress. Real-time RT-PCR analysis of selected genes showed by our microarray hybridization approach was capable of providing information about *S. cerevisiae* gene expression modulation with a considerably high level of confidence. Finally, a physical protein-protein (PPPI) network design and global topological analysis stressed the importance of these pathways in response of *S. cerevisiae* to propolis and were correlated with the transcriptional data obtained thorough the microarray analysis.

**Conclusions:**

In summary, our data indicate that propolis is largely affecting several pathways in the eukaryotic cell. However, the most prominent pathways are related to oxidative stress, mitochondrial electron transport chain, vacuolar acidification, regulation of macroautophagy associated with protein target to vacuole, cellular response to starvation, and negative regulation of transcription from RNA polymerase II promoter. Our work emphasizes again the importance of *S. cerevisiae* as a model system to understand at molecular level the mechanism whereby propolis causes cell death in this organism at the concentration herein tested. Our study is the first one that investigates systematically by using functional genomics how propolis influences and modulates the mRNA abundance of an organism and may stimulate further work on the propolis-mediated cell death mechanisms in fungi.

## Background

Propolis is a natural product of plant resins collected by honeybees (*Apis mellifera*) from various plant sources. It is used by the bees to seal holes in their honeycombs and protect the hive entrance [1-3]. Propolis has been used in folk medicine for centuries. Its chemical composition is quite complex since more than 300 compounds, such as polyphenols, phenolic aldehydes, sequiterpene quinines, coumarins, amino acids, steroids, and inorganic compounds, have been identified in propolis samples. Propolis has cytotoxic [4], anti-herpes virus [5], antitumor [6], radical scavenging [7], antimicrobial [8,9], antiprotozoan [10], and anti-HIV [11] activity and suppressive effects of dioxin toxicity [9]. More recently, evidence has shown that propolis can be used to treat *Candida* fungal infections [12-16].

Recently, we applied the power of *Saccharomyces cerevisiae* as a model organism for studies of genetics, cell biology, and genomics to determine how propolis affects eukaryotic cells at the cellular level [17]. Propolis is able to induce an apoptosis cell death response; however, increased exposure to propolis provides a corresponding increase in the necrosis response. We showed that cytochrome *c* but not endonuclease G (Nuc1p) is involved in propolis-mediated cell death in *S. cerevisiae*. We also observed that the metacaspase *YCA1* gene is important for propolis-mediated cell death. We screened the full collection of about 4,800 haploid *S. cerevisiae* non-essential deletion mutants for propolis sensitivity, and we were able to identify 138 deletion strains that have different degrees of propolis sensitivity compared to the corresponding wild-type strains. Our studies indicated that propolis sensitivity is dependent on the mitochondrial function and that vacuolar acidification and autophagy are important for yeast cell death caused by propolis. Here, we extended our understanding of propolis-mediated cell death in the yeast *Saccharomyces cerevisiae* by applying systems biology tools to analyze the transcriptional profiling of cells exposed to propolis.

## Methods

### Propolis tandardized extract

Propolis Standardized Extract - (EPP-AF®) (Batch 010/08) were industrially produced and kindly provided by Apis Flora Company (RibeirãoPreto/SP – Brazil). The extract was standardized using a propolis blend composed by raw material obtained from several sites of Brazil (Patent number PI 0405483–0, published in Revista de Propriedade Industrial n. 1778 from 01/02/2005). Propolis (blend of raw material) was kept in a freezer for 12 h, ground to a fine powder in a blender. It was then extracted using hydroalcoolic solution (7:3), with dinamic maceration, during 72 hours in ambient conditions (25° ± 5°C), followed by percolation process (1L/min.) and finally by a filtration process using in the first step the propolis biomass like a filter and secondly a 220 mesh stainless steel industrial line filter. Propolis extract obtained presents 11% w/v of dry matter and chemical composition standardized qualitatively and quantitatively by RP-HPLC (C18 Shim-pack, CLC-ODS (M), 25 cm x 4,6 column -with gradient elution with methanol and acidic water pH=2,7, plotted at 275 nm) into compounds: caffeic, *p*-coumaric and cinnamic acids, aromadendrin, isosakuranetin and artepillin C.

### Yeast strain, media and culture methods

The assays were carried out with *S. cerevisiae* yeast strain BY4742 (*MATα; his3*Δ*1; leu2*Δ*0; lys2*Δ*0; ura3*Δ*0*) [18]. The culture medium used was complete medium YPD (2% w/v glucose, 1% w/v yeast extract, 2% w/v peptone). For the microarray assay, the yeast cells were grown for 9 hours (mid-exponential phase) in 50mL of liquid YPD at 30°C with mechanical shaking (200rpm). After this, the cells (~ 2 x 10^7^ cells ml^-1^) were exposed to propolis 0.125% for 5 or 10 minutes. The control for the experiment using propolis 0.125% as a treatment has 0.68% ethanol. Two independent experiments were performed to each array using two different biological samples and dye swap analysis. Cell viability was determined by plating appropriate cell concentrations and counting the number of colonies in comparison to propolis-untreated controls.

### RNA isolation and real-time PCR

For total RNA isolation, the yeast cells were disrupted by vortexing with glass beads and total RNA was extracted with Trizol reagent (Invitrogen, USA). Ten micrograms of RNA from each treatment were then fractionated in 2.2 M formaldehyde, 1.2% w/v agarose gel, stained with ethidium bromide, and then visualized with UV-light. The presence of intact 25S and 18S ribosomal RNA bands was used as a criterion to assess the integrity of the RNA. RNAse free DNAse treatment was carried out as previously described [19]. After this, the total RNA was purification by RNeasy® Mini Kit (Qiagen) and the purified samples were measured in the NanoDrop® 2000 (Thermo Scientific).

### Microarray hybridization

For gene expression analysis commercially-available Agilent whole genome *S. cerevisiae* microarray [Yeast (V2) Gene Expression Microarray, 8x15K] was used. The microarray slides contain 15,208 probes for *S.cerevisiae* (BY4742 strain). The RNA samples obtained under the conditions above described were subjected to cRNA fluorescent labeling. cRNA labeling was performed according to the standard protocol described by Agilient using Two-Color Microarray-Based Gene Expression Analysis (Agilent Technologies, USA). Briefly, for cRNA synthesis and labeling 5 μg of total RNA were used. After labeling, 300 ng of Cy3 and Cy5-labelled cRNAs (specific activity > 8.0 pmol Cy3-Cy5/μg cRNA) was fragmented at 60°C for 30 minutes in a reaction volume of 25 μl containing 1x Agilent fragmentation buffer and 2x Agilent blocking agent following the manufacturer’s instructions. On completion of the fragmentation reaction, 25 μl of 2x Agilent hybridization buffer was added to the fragmentation mixture and hybridized to the *S. cerevisiae* microarrays slides for 17 hours at 65°C in an Agilent G2545A Hybridization Oven and on Agilent Rotator Rack. After hybridization, microarrays were sequentially washed: 1 minute at room temperature with GE Wash Buffer 1 (Agilent) and 1 minute with 37°C GE Wash buffer 2 (Agilent), then a 10 seconds Acetonitrile Wash (Agilent) followed by a 30 seconds Stabilization and Drying Solution wash (Agilent). Slides were immediately subjected to fluorescent detection using fluorescent detection with a GenePix 4000B (Molecular Devices, USA) with simultaneously scanning the Cy3 and Cy5 channels at a resolution of 5 μm. Laser was set at 100% and PMT gain was adjusted automatically for each slide using the program GenePix Pro (Molecular Device) according to the signal intensity of each array. Merged Cy3 and Cy5 TIFF images generated by the GenePix Pro were used to analysis in the Agilent Feature Extraction software (version 9.5.3.1, Agilent) using Linear Lowess algorithm to obtain background subtracted and normalized intensity values. The dye-normalyzed values generated in the Feature Extraction data files were used to upload the software Express Converter (version 2.1, TM4 available at http://www.tm4.org/utilities.html) which conveniently converts the Agilent file format to mev (multi experiment view) file format compatible to the TM4 softwares for microarray analysis (available at http://www.tm4.org/). The mev files were then uploaded in the MIDAS software where the resulting data were averaged from replicated genes on each array, from dye-swap hybridizations for each experiment and from two biological replicates using the tools “flip dye consistency cheking” and “in slides replicates analysis” implemented in MIDAS software. The mev files generated were then loaded in MEV software (MultiExperiment Viewer) where differentially expressed genes were identified using one-class *t*-test (p>0.01). Significantly different genes were those whose mean log_2_ expression ratio over all included samples was statistically different from 0 which indicates the absence of gene modulation. The genes significantly up- or down-regulated in the microarray analysis was subjected to Gene Ontology analysis using the GO Term Finder tool available at the *Saccharomyces* Genome Database (SGD <http://www.yeastgenome.org>).

### Physical protein-protein (PPPI) network design and global topological analysis

The transcriptomic data gathered from yeast BY4742 strain submitted to propolis treatment was used to obtain information about how the under- and overexpressed genes and their products interact in the context of physical protein-protein interactions (PPPI networks) in *S. cerevisiae.* In this sense, the data mining screening and network design of repressed or induced genes-associated PPPI networks was performed using Cytoscape software, version 2.6.3 [20]. For this purpose, we used the PPPI data of *S. cerevisiae* available in the *Saccharomyces* Genome Database ( http://www.yeastgenome.org). The induced and repressed PPPI networks obtained from this first screening were then combined in a unique PPPI network by employing the union function of the Cytoscape core plugin Merge Networks. The union PPPI network was then analyzed with molecular complex detection (MCODE) software [21], a Cytoscape plug-in (at http://apps.cytoscape.org/apps/mcode) in order to detect clusters of proteins that could represent distinct biologic processes. The parameters used for MCODE to generate the sub networks were as follows: loops included; degree cutoff of 2; deletion of single connected nodes from cluster (haircut option enabled); expansion of cluster by one neighbor shell allowed (fluff option enable); node density cutoff of 0.1; node score cutoff of 0.2; *k*-core of 2; and maximum depth of network of 100. The degree of data overlapping between induced- and repressed-associated PPPI networks was obtained from an area-proportional Venn diagram analysis, available at <http://bioinforx.com/free/bxarrays/overlap.php>.

### Network centralities and local topological analyses

Two major network centralities (node degree and betweenness) were computed from the merged network and clusters using the Cytoscape plugin CentiScaPe 1.0 [22]. The local topology of the network, defined as bottlenecks, was obtained from the threshold generated by each centrality calculated by CentiScape 1.0. In this sense, bottlenecks were defined as nodes with a value above the threshold calculated for node degree and betweenness.

### Gene ontology analysis

Gene ontology (GO) clustering analysis was performed using Biological Network Gene Ontology (BiNGO) [23] software, a Cytoscape plugin available at http://chianti.ucsd.edu/cyto_web/plugins/index.php. The degree of functional enrichment for a given cluster and category was quantitatively assessed (*p* value) by hypergeometric distribution [24] and a multiple test correction was applied using the false discovery rate (FDR) [25] algorithm, fully implemented in BiNGO software. Overrepresented biological process categories were generated after FDR correction, with a significance level of 0.05.

## Results and discussion

### Microarray hybridization analysis

To our knowledge, previous to our work there is only a single study in the literature reporting transcriptional profiling for eukaryotic cells exposed to propolis [26]. In this study, propolis was applied for 24 hours to normal human dermal fibroblast and keratinocytes. These authors were able to identify 205 genes important for skin and only 5 (ATP citrate synthase, aquaporin 3, cytochrome *c* oxidase 1, nitric oxide synthase 3, and hydroxylase 3) and 1 (progestone receptor) that appear to be up- and down regulated in both cell lines, respectively. We have been using *S. cerevisiae* as an eukaryotic model system to identify genes that are important for propolis-mediated cell death. As previously shown, when *S. cerevisiae* exponential cells are exposed to propolis 0.125% for 5 and 10 minutes, there is a decreased survival of 24.1 and 6.3%, respectively in comparison to the propolis-untreated control containing only 0,68% ethanol [17]. To gain an insight on which pathways are modulated during *S. cerevisiae* exposure to propolis, we determined its transcriptional profiling. Total RNA extracted from these cultures was used to generate fluorescent-labeled cRNAs for competitive microarray hybridizations. All the controls for further experiments using propolis 0.125% as a treatment have 0.68% ethanol (reference samples). We have compared the mRNA expression of the *S. cerevisiae* BY4742 strain grown for 9 hours and exposed to 0.125% propolis for 5 and 10 minutes with yeast cells exposed to 5 and 10 minutes 0.68% ethanol. In these experiments, the main aim was to focus on genes that have increased or decreased mRNA expression. The full dataset was deposited in the Gene Expression Omnibus (GEO) from the National Center of Biotechnology Information (NCBI) with the number GSE33971 ( http://www.ncbi.nlm.nih.gov/projects/geo/query/acc.cgi?acc=GSE33971). We were able to observe 1,399 genes modulated in at least one time point (*p-*value 0.01, calculated FDR is 2.1%) when compared to the respective reference time (reference untreated samples obtained under the control experimental conditions, i. e., 2 x 10^7^ cells ml^-1^ exposed only to 0.68% ethanol for 5 or 10 minutes). We have used Gene Ontology (GO) Term Finder analysis aiming to classify the main biological processes associated to the list of the up- and down-regulated genes identified in the microarray hybridizations. In addition, we assessed the probability values of the over-abundance of the GO groups compared to the genomic average in order to gain information about the statistical significance of overrepresented processes (*p*<0.05). Table 1 shows the adjusted *p*-values indicating the categories of genes overrepresented in the microarray analysis which are involved in a variety of cellular processes. To have a broader view of the most significant modulated genes found in the microarray analysis, we listed the genes having increased or decreased mRNA expression with log ratios ≥ 1 (203 genes) or ≤ 1 (136 genes). These genes were grouped according to the GO identity obtained in Table 1 [Additional file 1: Table A1 shows the genes with log ratios ≥ 1 (203 genes) or ≤ 1 (136 genes), respectively]. The Table 2 shows a list of chosen genes presenting higher level of up-regulation in the microarray which were grouped into categories of significantly overrepresented biological process according to the gene ontology ID shown in Table 1. Interestingly, inside the enriched category “transmembrane transport and localization” (GO:0055085 and GO:0051179; *p*<0.01), we have observed several genes encoding transporters reported as involved in multidrug resistance (MDR; for reviews see [27-30], suggesting propolis can activate at the transcriptional level the complex set of genes responsible for MDR in *S. cerevisiae* (Table 2 and Additional file 1: Table A1). In addition, we have also observed enrichment for genes encoding proteins important for the assembly of the vacuolar ATPase and the endocytic pathway (Table 2 and Additional file 1: Table A1). None of the deletion mutants for these transporter encoding genes shown in Additional file 1: Table A1 were observed as more sensitive to propolis, except for *TPO1*[17], indicating a redundant transcriptional response of these genes to propolis. However, we have previously observed that when several genes involved in the assembly of the yeast V-ATPase such as *VPH1*, *VMA3, 4, 5, 11, 22*, *RAV1* and *SOP4* were deleted, the corresponding yeast deletion strains became more sensitive to propolis [17].

**Table 1:** Overrepresented categories of the significantly modulated (up- and down-regulated) genes found in the microarray hybridization based on the genome coverage (p<0.05)

**GOID**	**TERM**	**CORRECTED *P*-VALUE**	**FDR RATE**
GO:0009987	cellular process	3.57E-09	0.00%
GO:0065007	biological regulation	7.91E-09	0.00%
GO:0050794	regulation of cellular process	1.58E-07	0.00%
GO:0050789	regulation of biological process	7.16E-07	0.00%
GO:0051276	chromosome organization	1.30E-06	0.00%
GO:0006996	organelle organization	2.25E-06	0.00%
GO:0071841	cellular component organization or biogenesis at cellular level	2.66E-06	0.00%
GO:0071842	cellular component organization at cellular level	3.14E-06	0.00%
GO:0022402	cell cycle process	3.16E-06	0.00%
GO:0007049	cell cycle	4.76E-06	0.00%
GO:0071840	cellular component organization or biogenesis	5.08E-06	0.00%
GO:0022403	cell cycle phase	5.56E-06	0.00%
GO:0006351	transcription, DNA-dependent	1.13E-05	0.00%
GO:0032774	RNA biosynthetic process	1.28E-05	0.00%
GO:0016043	cellular component organization	1.95E-05	0.00%
GO:0000279	M phase	1.98E-05	0.00%
GO:0007059	chromosome segregation	2.94E-05	0.00%
GO:0010556	regulation of macromolecule biosynthetic process	3.70E-05	0.00%
GO:0060255	regulation of macromolecule metabolic process	4.61E-05	0.00%
GO:0019219	regulation of nucleobase-containing compound metabolic process	4.64E-05	0.00%
GO:2000112	regulation of cellular macromolecule biosynthetic process	4.86E-05	0.00%
GO:0051171	regulation of nitrogen compound metabolic process	4.96E-05	0.00%
GO:0006807	nitrogen compound metabolic process	6.46E-05	0.00%
GO:0051252	regulation of RNA metabolic process	0.000109266	0.00%
GO:0034641	cellular nitrogen compound metabolic process	0.000114855	0.00%
GO:0006325	chromatin organization	0.00011847	0.00%
GO:0009889	regulation of biosynthetic process	0.00012496	0.00%
GO:0031326	regulation of cellular biosynthetic process	0.00012496	0.00%
GO:0000278	mitotic cell cycle	0.000140145	0.00%
GO:0006355	regulation of transcription, DNA-dependent	0.000140184	0.00%
GO:2001141	regulation of RNA biosynthetic process	0.000140184	0.00%
GO:0048285	organelle fission	0.00015597	0.00%
GO:0007067	mitosis	0.000167729	0.00%
GO:0000280	nuclear division	0.000263661	0.00%
GO:0080090	regulation of primary metabolic process	0.000279393	0.00%
GO:0006139	nucleobase-containing compound metabolic process	0.000384242	0.00%
GO:0065003	macromolecular complex assembly	0.001007045	0.00%
GO:0031323	regulation of cellular metabolic process	0.00115049	0.00%
GO:0000087	M phase of mitotic cell cycle	0.001853624	0.00%
GO:0019222	regulation of metabolic process	0.002020633	0.00%
GO:0043933	macromolecular complex subunit organization	0.002159081	0.00%
GO:0050896	response to stimulus	0.002997939	0.00%
GO:0051179	localization	0.003108437	0.00%
GO:0033043	regulation of organelle organization	0.00356682	0.00%
GO:0048523	negative regulation of cellular process	0.003751468	0.00%
GO:0010468	regulation of gene expression	0.004560142	0.00%
GO:0016568	chromatin modification	0.006683962	0.00%
GO:0048519	negative regulation of biological process	0.006744751	0.00%
GO:0034622	cellular macromolecular complex assembly	0.007563808	0.00%
GO:0006950	response to stress	0.009479328	0.00%
GO:0055085	transmembrane transport	0.011885843	0.00%
GO:0006366	transcription from RNA polymerase II promoter	0.01881881	0.00%
GO:0010604	positive regulation of macromolecule metabolic process	0.021216691	0.00%
GO:0034621	cellular macromolecular complex subunit organization	0.024007449	0.00%
GO:0016070	RNA metabolic process	0.026116229	0.00%
GO:0009058	biosynthetic process	0.028171132	0.00%
GO:0051173	positive regulation of nitrogen compound metabolic process	0.032491519	0.00%
GO:0000819	sister chromatid segregation	0.033192255	0.00%
GO:0009893	positive regulation of metabolic process	0.034622731	0.00%
GO:0031325	positive regulation of cellular metabolic process	0.035672319	0.00%

Enrichment analysis was performed using the GO Term Finder available at <http://go.princeton.edu/cgi-bin/GOTermFinder> for searching for significant shared GO terms to describe the gene list of the significantly modulated genes (p<0.05). The input list for the analysis comprised 1,399 representing the total of up-and down-regulated found in the microarray analysis. FDR is the percentage of the GO terms with p-values as good as or better than a particular GO term with this FDR would be expected to be false positives.

**Table 2:** **Selected genes more expressed (log2 ≥ 1.0) during *S. cerevisiae *exposure to propolis according to the GO term finder enrichment analysis (for a complete list of the genes more expressed, see Additional file 1: Table A1)**

**Transmembrane Transport (GO:0055085) and Localization (GO:0051179) (p*<*0.011)**	
FLR1	Multidrug transporter of the MFS, involved in efflux of fluconazole, diazaborine, benomyl, methotrexate, and other drugs
PDR15	ATP binding cassette (ABC) transporter, multidrug transporter and general stress response factor implicated in cellular detoxification
AZR1	Transporter of the MFS, involved in resistance to azole drugs such as ketoconazole and fluconazole
YOR1	ATP-binding cassette (ABC) transporter, multidrug transporter mediates export of many different organic anions including oligomycin
PDR10	ATP-binding cassette (ABC) transporter, multidrug transporter involved in the pleiotropic drug resistance network
PDR12	ATP-binding cassette (ABC) transporter, weak-acid-inducible multidrug transporter required for weak organic acid resistance
ENA1	P-type ATPase sodium pump, involved in Na+ and Li+ efflux to allow salt tolerance
ENA2	P-type ATPase sodium pump, involved in Na+ efflux to allow salt tolerance; likely not involved in Li+ efflux
VMA21	Membrane protein that is required for vacuolar H+−ATPase (V-ATPase) function, although not an actual component of the V-ATPase
TPO1	Polyamine transporter (MFS) that recognizes spermine, putrescine, and spermidine
TPO4	Polyamine transport protein (MFS) recognizes spermine, putrescine, and spermidine
*ATG22*	Vacuolar integral membrane protein required for efflux of amino acids during autophagic body breakdown in the vacuole
VMA7	Subunit F of the eight-subunit V1 peripheral membrane domain of vacuolar H+−ATPase (V-ATPase)
PKR1	V-ATPase assembly factor, functions with other V-ATPase assembly factors in the ER to efficiently assemble the V-ATPase
**Response to Stress (GO:0006950) and Response to Stimulus (GO:0050896) (p<0.009)**	
GRX4	Hydroperoxide and superoxide-radical responsive glutathione-dependent oxidoreductase
*GTT3*	Protein of unknown function with a possible role in glutathione metabolism
TSA2	Stress inducible cytoplasmic thioredoxin peroxidase; cooperates with Tsa1p in the removal of reactive oxygen
GTT2	Glutathione S-transferase capable of homodimerization; functional overlap with Gtt2p, Grx1p, and Grx2p
GND2	6-phosphogluconate dehydrogenase, catalyzes an NADPH regenerating reaction in the pentose phosphate pathway
TRX1	Cytoplasmic thioredoxin isoenzyme of the thioredoxin system which protects cells against oxidative and reductive stress

Earlier, by screening a non essential yeast deletion library, we observed that most of the proteins whose deletion increases the sensitivity of yeast strains to propolis are involved in cell division mechanisms, mitochondrial electron transport chain, vacuolar acidification, regulation of macroautophagy associated with protein target to vacuole, cellular response to starvation, and negative regulation of transcription from RNA polymerase II promoter [17]. We have shown that propolis induces vacuolar acidification and translocation of Atg8p to the vacuoles, one of the hallmarks of autophagy [17]. In *S. cerevisiae*, the vacuole is very important for keeping cellular homeostasis comprising the regulation of intracellular pH and degradation mainly during nutrient limitation of proteins and organelles by autophagy (for reviews, see [31-33]). Cell death induced by acetic acid is increased in *S. cerevisiae VPS* gene deletion mutants (*VPS* genes are involved in homotypic vacuole fusion, vacuolar protein sorting and are essential for normal vacuolar function) [34]. It has been observed that the intracellular pH was acidified in *VPS* mutant cells upon treatment with acetic acid [34]. It is possible the disturbance of the homeostatic pH control may trigger necrosis by release of pro-necrotic proteases, which would find an optimal pH for their enzymatic activity in the acidified cytosol [35]. We have observed genes encoding proteins important for ROS detoxification in *S. cerevisiae* significantly enriched in the microarray hybridization (GO: GO:0006950; Response to Stress and (GO:0050896) Response to Stimulus; Table 2 and Additional file 1: A1; p<0.003) such as *GRX4*, *GTT2, GTT3, TSA2, DFM1,* and *TRX1* with increased mRNA accumulation when *S. cerevisiae* is exposed to 0.125% propolis. Interestingly, there is also an increased mRNA accumulation of genes encoding proteins involved in the generation of ATP into the mitochondria, such as *ATP17*, *ATP18*, *ATP19*, *ATP20*, *ATP21*, and *COX8* grouped in the category of cellular component organization or biogenesis at cellular level (GO:0071841; Table 2 and Additional file 1: Table A1; *p* < 2.66 x10^-6^). There are several conditions where mitochondria-produced ROS have been associated to yeast apoptosis (for reviews, see [35-38]). Propolis at 0.125% can induce ROS formation and it is more lethal when *S. cerevisiae* grows in the presence of glycerol and ethanol as carbon sources [17], suggesting that respiration increases propolis lethality. Interestingly, it was observed as up-regulated into the same enriched category the gene *OYE3* (old yellow enzyme) which was described as involved in increased resistance to H_2_O_2_-induced programmed cell death in yeast [39].

Moreover, there are several genes related to cell cycle and cell cycle process and chromosome distribution and chromatin silencing that have decreased mRNA accumulation when *S. cerevisia*e is exposed to propolis (Table 3 and Additional file 1: Table A1). The reduced mRNA abundance of these genes suggested propolis is activating transcriptional checkpoint controls involved in the S- and M-phases important for DNA replication and proper chromosome segregation.

**Table 3:** **Selected genes less expressed (log2 ≤ −1.0) during *S. cerevisiae *exposure to propolis according to the GO term finder enrichment analysis (for a complete list of the genes less expressed, see Additional file****1****: Table A1)**

**Cell cycle (GO:0007049) and Cell cycle process (GO: 0022402) (*p*< 4.76x10**^**-6**^**)**	
SMC4	Subunit of the condensin complex; reorganizes chromosomes during cell division
*MCM10*	Essential chromatin-associated protein involved in the initiation of DNA replication
CIN8	Kinesin motor protein involved in mitotic spindle assembly and chromosome segregation
IBD2	Component of the BUB2-dependent spindle checkpoint pathway, interacts with Bfa1p and functions upstream of Bub2p and Bfa1p
BRN1	Subunit of the condensin complex; required for chromosome condensation and for clustering of tRNA genes at the nucleolus
CEP3	Essential kinetochore protein, component of the CBF3 complex that binds the CDEIII region of the centromere
SLK19	Kinetochore-associated protein required for normal segregation of chromosomes in meiosis and mitosis
NSL1	Component of the MIND kinetochore complex which joins kinetochore subunits contacting DNA to those contacting microtubules
SPC24	Component of the evolutionarily conserved kinetochore-associated Ndc80 complex
HOS2	Histone deacetylase required for gene activation via specific deacetylation of lysines in H3 and H4 histone tails
ESA1	Catalytic subunit of the histone acetyltransferase complex (NuA4) that acetylates four conserved internal lysines of histone H4
MAM1	Monopolin, kinetochore associated protein involved in chromosome attachment to meiotic spindle
**Chromosome organization (GO:0051276) (p< 1.30x10**^**-6**^**)**	
RLF2	Largest subunit (p90) of the Chromatin Assembly Complex (CAF-1) and Msi1p that assembles newly synthesized histones
SGF29	Probable subunit of SAGA histone acetyltransferase complex
**RNA metabolic process (GO:0016070) and RNA biosynthetic process (GO:0009058) (p< 0.02)**	
LEO1	Component of the Paf1 complex, which associates with RNA polymerase II and is involved in histone methylation
SPT21	Protein required for normal transcription at several loci including HTA2-HTB2 and HHF2-HHT2
*RFM1*	Specificity factor that directs the Hst1p histone deacetylase to some of the promoters regulated by Sum1p
IES2	Protein that associates with the INO80 chromatin remodeling complex under low-salt conditions
IFH1	IFH1 exhibits genetic interactions with FHL1, overexpression interferes with silencing at telomeres and HM loc
NGG1	component of transcriptional adaptor and histone acetyltransferase complexes, the ADA, the SAGA, and the SLIK complexes

### Validation of the microarray hybridization analysis

**Figure 1:**
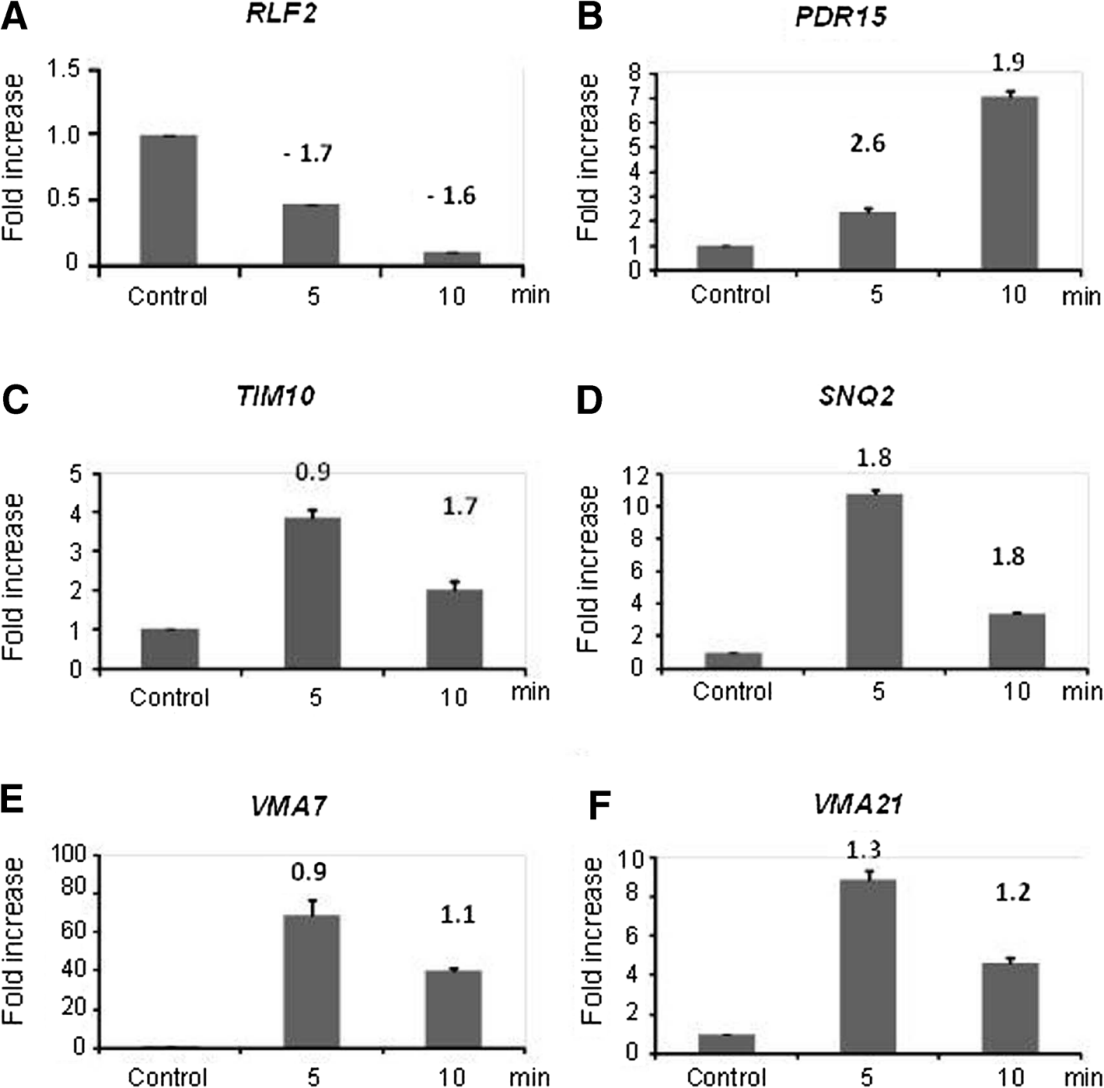
**Real-time RT-PCR for selected genes from the microarray hybridization analysis**. *S. cerevisiae* was grown for 9 hours in liquid YPD at 30°C and the cells (~ 2 x 10^7^ cells ml^-1^) were transferred to fresh liquid YPD and exposed to propolis 0.125% for 5 or 10 minutes. The relative quantitation of *RLF2* (**A**), *PDR15* (**B**), *TIM10* (**C**), *SNQ2* (**D**), *VMA7* (**E**), *VMA21* (**F**) was performed using *TAF10* as normalizer. Gene expression was determined by a standard curve (i.e., C_T_ –values plotted against logarithm of the DNA copy number). The results are the means ± standard deviation of four sets of experiments using completely independent biological replicates. The values above the bars are mean of the log2-ratio obtained in the microarray hybridization experiments.

To validate some of our findings, we have chosen six different genes from our microarray analysis whose mRNA has either increased or decreased abundance when *S. cerevisiae* is exposed to 0.125% propolis. We designed Lux fluorescent probes and used real-time RT-PCR analysis to quantify their expression in a new set of biological replicate of the mRNA isolated from 5 and 10 minutes exposure to 0.125% propolis and compared them with the corresponding 5 and 10 minutes exposure to 0.68% ethanol. We have used as a normalizer control, *TAF10*, a gene encoding a subunit (145 kDa) of TFIID and SAGA complexes, involved in RNA polymerase II transcription initiation and in chromatin modification. Recently, this gene was shown as an appropriate reference gene for quantitative gene expression analysis by real-time RT-PCR [40]. In addition, *TAF10* was not shown as modulated in our microarray hybridization experiments (data not shown). Thus, the measured quantity of a specific gene mRNA in each of the treated samples was normalized using the C_T_ values obtained for the *TAF10* mRNA amplifications run in the same plate. The results were expressed as the number of times the genes have increased or decreased abundance when the yeast strains were exposed to propolis compared to the ethanol treatment (Figure 1).

Due to the apparent importance of the enriched genes in the categories of (i) cellular component organization or biogenesis; (ii) transmembrane transport; (iii) response to stress/stimulus and (iv) cellular component organization or biogenesis, we evaluated the mRNA abundance of (i) *PDR15* (YDR406W) encoding a transporter involved in multidrug resistance; (ii) *SNQ2* (YDR011W) encoding a plasma membrane ATP-binding cassette (ABC) transporter, multidrug transporter involved in multidrug resistance and resistance to singlet oxygen species; (iii) *TIM10* (YHR005C-A) encoding and essential protein of the mitochondrial intermembrane space, that forms a complex with Tim9p (TIM10 complex) and delivers hydrophobic proteins to the TIM22 complex for insertion into the inner membrane; (iv) *VMA7* (YGR020C) and (v) *VMA21* (YGR105W). These genes respectively encodes the subunit F of the eight-subunit V1 peripheral membrane domain of vacuolar H^+^-ATPase (V-ATPase) and Integral membrane protein that is required for vacuolar H+−ATPase (V-ATPase) function. The gene *RLF2* (YPR018W) was also analyzed but different from the previous chosen genes, *RLF2* was observed as down-regulated in the microarray analysis (Additional file 1: Table A1; Chromosome organization (GO:0051276; *p*<1.3 x10^-6^). *RLF2* encodes the largest subunit (p90) of the Chromatin Assembly Complex (CAF-1) with Cac2p and Msi1p that assembles newly synthesized histones onto recently replicated DNA.

As expected, all five genes that showed increased mRNA abundance (*PDR15*, *TIM10*, *SNQ2*, *VMA7*, and *VMA21*) and decreased mRNA abundance (*RLF2*) in the microarray hybridization analysis grouped in its respective enrichment categories showed corresponding increase and decreased expression in the real-time PCR experiments (Figure 1A-F). This behavior is in accordance with the normalized mean values obtained in the microarray analysis (see values above the Figure 1 graphs for comparison). Thus, it seems that our microarray hybridization approach is capable of providing information about *S. cerevisiae* gene expression modulation with a considerably high level of confidence and is an open source of data for further investigation for the mechanisms of propolis-mediated cell death in all susceptive organisms.

### Systems analysis for propolis exposure

**Figure 2:**
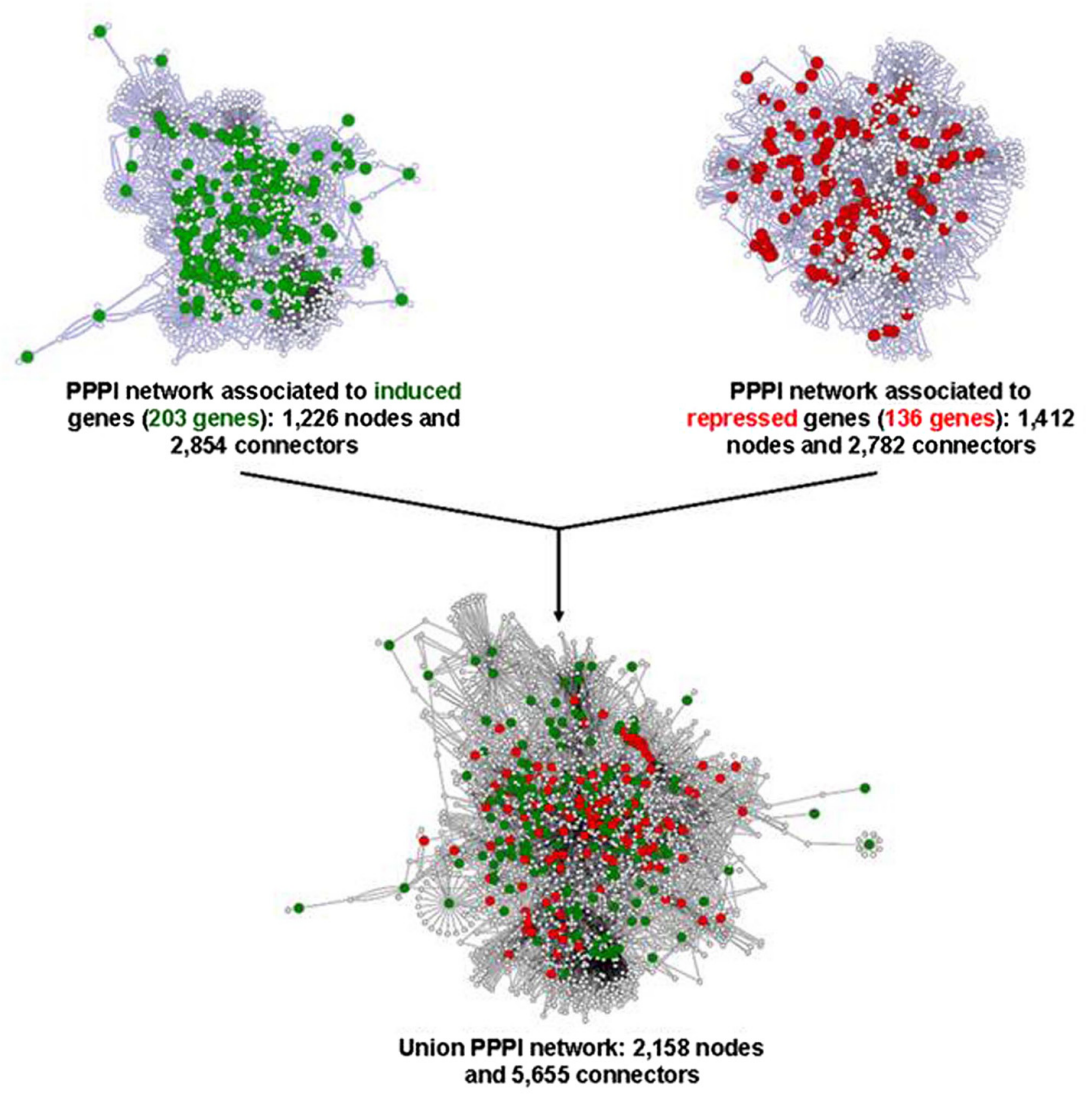
**Design of the union network generated from repressed- and induced-associated gene PPPI networks**. Green nodes indicate proteins of genes that were induced in the array, while red nodes are associated to proteins of genes that were repressed in the array.

The transcriptomics data obtained submitted to the treatment conditions described in this work prompt us to ask how the underexpressed or overexpressed genes affect different biological processes that are altered during the exposure to 0.125% propolis. In this sense, a search for potential proteins and/or mechanisms and their associated biological processes that are affected by the conditions treatment was initiated. To achieve this goal, two different PPPI networks using yeast transcriptomics data were retrieved from *Saccharomyces* Genome Database (SGD): one associated to repressed genes (136 genes; repressed genes-associated PPPI network) and one associated to induced genes (203 genes; induced genes-associated network). The induced genes-associated PPPI network obtained from SGD contains 1,226 nodes and 2,854 connectors while the repressed-associated gene PPPI network contains 1,412 nodes and 2,782 connectors (Additional file 2: Table A2). Both induced- and repressed-genes associated PPPI networks were analyzed in order to observe the degree of network overlapping by means of an Area proportional Venn diagram. This analysis indicated that the repressed-genes associated PPPI network contains 919 unique proteins, while the induced-genes associated network contains 733 unique proteins, and there are 493 overlapping proteins. Although the degree of network overlapping was not elevated, we decided to merge both networks in a unique graph, containing 2,158 nodes and 5,655 connectors (Figure 2 and Additional file 2: Table A2). Sub networks (clusters) present in the union PPPI network were identified and retrieved using the Cytoscape-associated plugin MCODE and subjected to a Gene Ontology (GO) analysis in order to obtain information about the nature and number of sub graphs belonging to the network and their associated biological processes. The union PPPI network contains eleven interconnected clusters, each comprising different biological processes. GO analysis of the obtained clusters indicated the participation of important biological processes that can be seen in Table 4 and Additional file 3: Table A3. Many of these processes are reflecting the results previously found in the yeast library screening such as cell division mechanisms, mitochondria, vacuolar acidification, negative regulation of transcription from RNA polymerase II promoter (17). In addition, other important processes such as protein transport and membrane organization and biogenesis (Table 4, cluster 3); transcription from RNA polymerase II promoter and transcription, DNA dependent (Table 4, cluster 4), regulation of cell cycle (Table 4, cluster 7); ATP metabolic process, proton transport and vacuolar acidification (Table 4, cluster 9) and response to stress (Table 4, cluster 10) were categories also observed in the microarray hybridization analysis (see Additional file 3: Table A3 for a complete description of genes). It is also important to mention that statistically significant modulated genes could be observed in each of these clusters indicating that the PPPI network could link the transcriptomic analysis to potential biological processes affected by propolis. These genes can be seen in Additional file 3: Table A3 [written in red (repressed) or green (induced)].

**Table 4:** Main specific gene ontology categories observed in clusters derived from union PPPI network

	**Biological process**	**GOID**	***P*-value**^**a**^	**Corrected P value**^**b**^	**k**^**c**^	**ƒ**^**d**^
**Cluster 1**	Translation	6416	2.07 x 10^-45^	2.93 x 10^-43^	45	499
	Ribosome assembly	42255	1.82 x 10^-9^	2.58 x 10^-8^	9	65
	Negative regulation of mRNA processing	50686	8.25 x 10^-3^	3.90 x 10^-2^	1	1
**Cluster 2**	Organic acid transport	15849	2.0594 x 10^-4^	1.4107 x 10^-2^	3	65
	Choline transport	15871	1.8904 x 10^-3^	3.2372 x 10^-2^	1	1
	Betaine transport	15838	1.8904 x 10^-3^	3.2372 x 10^-2^	1	1
	Ethanolamine transport	34229	1.8904 x 10^-3^	3.2372 x 10^-2^	1	1
	Amino acid transport	6865	3.4951 x 10^-3^	4.7047 x 10^-2^	2	48
**Cluster 3**	Mitochondrial transport	6839	1.74 x 10^-10^	3.88 x 10^-9^	5	67
	Mitochondrion organization and biogenesis	7005	2.6080 x 10^-8^	2.9122 x 10^-7^	5	179
	Protein transport	15031	3.7859 x 10^-6^	2.1138 x 10^-5^	5	481
	Membrane organization and biogenesis	16044	3.5801 x 10^-4^	1.0903 x 10^-3^	3	196
**Cluster 4**	Transcription from RNA polymerase II promoter	6366	3.4996 x 10^-11^	3.4296 x 10^-9^	9	162
	Histone modification	16570	1.1400 x 10^-9^	2.2345 x 10^-8^	7	92
	Establishment and/or maintenance of chromatin architecture	6325	2.4536 x 10^-9^	4.3719 x 10^-8^	9	260
	Transcription, DNA-dependent	6351	5.8940 x 10^-9^	8.7746 x 10^-8^	9	287
	G1 phase of mitotic cell cycle	80	1.0490 x 10^-8^	1.2850 x 10^-7^	5	32
	Regulation of RNA metabolic process	51252	1.5270 x 10^-8^	1.5753 x 10^-7^	11	614
	Chromosome organization and biogenesis	7001	1.0393 x 10^-7^	8.8567 x 10^-7^	9	398
	Response to drug	17035	3.3259 x 10^-3^	1.1436 x 10^-2^	3	121
	Cell cycle	7049	1.1244 x 10^-2^	3.5546 x 10^-2^	5	566
**Cluster 5**	DNA catabolic process	6308	7.6846 x 10^-5^	6.1477 x 10^-4^	2	30
	Meiotic recombination	7145	2.8107 x 10^-4^	1.6864 x 10^-3^	2	57
	Double-strand break repair	6302	3.0125 x 10^-4^	1.7012 x 10^-3^	2	59
	M phase of meiotic cell cycle	51327	2.6749 x 10^-3^	7.7814 x 10^-3^	2	176
	Telomere maintenance via recombination	722	9.7652 x 10^-3^	2.5337 x 10^-2^	1	19
**Cluster 6**	ER to Golgi vesicle-mediated transport	6888	6.1201 x 10^-4^	2.5773 x 10^-2^	2	84
**Cluster 7**	Negative regulation of RNA metabolic process	51253	2.4984 x 10^-6^	8.0002 x 10^-5^	4	157
	Negative regulation of transcription	16481	3.1251 x 10^-6^	8.0002 x 10^-5^	4	166
	Heterochromatin formation	31507	3.7388 x 10^-5^	2.9910 x 10^-4^	3	92
	Chromatin silencing	6342	3.7388 x 10^-5^	2.9910 x 10^-4^	3	92
	Gene silencing	16458	5.4046 x 10^-5^	3.8433 x 10^-4^	3	104
	Chromatin modification	16568	5.8205 x 10^-4^	2.8655 x 10^-3^	3	231
	Regulation of cell cycle	74	7.1144 x 10^-3^	1.5701 x 10^-2^	2	160
**Cluster 8**	RNA biosynthetic process	32774	2.4585 x 10^-3^	1.8302 x 10^-2^	2	289
	Transcription	6350	1.0545 x 10^-2^	4.8996 x 10^-2^	2	598
**Cluster 9**	Intracellular pH reduction	51452	4.8791 x 10^-5^	6.6815 x 10^-4^	2	24
	Vacuolar acidification	7035	4.8791 x 10^-5^	6.6815 x 10^-4^	2	24
	Regulation of intracellular pH	51453	5.3028 x 10^-5^	6.6815 x 10^-4^	2	25
	Proton transport	15992	1.1756 x 10^-4^	9.1538 x 10^-4^	2	37
	Cellular homeostasis	19725	2.1588 x 10^-3^	6.8004 x 10^-3^	2	158
	Ion transport	6811	2.3248 x 10^-3^	6.9745 x 10^-3^	2	164
	ATP metabolic process	46034	1.2324 x 10^-2^	2.9858 x 10^-2^	1	24
**Cluster 10**	Histone methylation	16571	1.2655 x 10^-9^	1.0504 x 10^-7^	5	17
	Regulation of transcription	45449	5.8795 x 10^-8^	2.0914 x 10^-6^	12	631
	Post-translational protein modification	43687	3.6875 x 10^-7^	3.9921 x 10^-6^	10	454
	Chromosome organization and biogenesis	7001	1.7266 x 10^-5^	9.3463 x 10^-5^	8	398
	Regulation of conjugation with cellular fusion	31137	4.4395 x 10^-3^	1.3481 x 10^-2^	2	31
	Ethanol biosynthetic process during fermentation	43458	6.5202 x 10^-3^	1.7647 x 10^-2^	1	2
	Response to stress	6950	1.2590 x 10^-2^	2.9574 x 10^-2^	6	632
	Glycolytic fermentation	19660	2.2645 x 10^-2^	4.6218 x 10^-2^	1	7
**Cluster 11**	Chromosome organization and biogenesis	7001	1.1865 x 10^-3^	2.9377 x 10^-2^	5	398
	DNA repair	6281	1.2413 x 10^-3^	2.9377 x 10^-2^	4	228
	Mitotic cell cycle	278	3.2147 x 10^-3^	4.5671 x 10^-2^	4	295
	Histone acetylation	16573	3.4260 x 10-3	4.5671 x 10^-2^	2	40

^*a*^*P* values were calculated by the hypergeometric distribution of one ontology class visualized in the network.

^*b*^ Calculated values based on *P* values obtained after FDR was applied.

^*c*^ Total number of proteins found in the network which belong to a gene ontology.

^*d*^ Total number of proteins that belong to a specific gene ontology.

Based on data gathered from this initial systems biology tools analysis, we decided to get more information about the major nodes involved in the information of the network using network centralities. Network centralities allow us to identify nodes (and the consequent biological processes) that have a relevant position in the overall network architecture [41]. Centralities have been recently applied to quantify the centrality and prestige of actors in social networks [41] and to understand the structure and properties of complex biological, technological and infrastructural networks [42,43]. Many of the nodes in a given network that show elevated values of centrality are important points of vulnerability, indicating that any attack to these nodes could introduce strong perturbations in the network. Node degree represents the simplest centrality measure in a given network, corresponding to the number of nodes adjacent to a given node, where adjacent means directly connected [22]. The node degree represents the “popularity” of a given node, and highly connected nodes in a network are termed hubs. Next, betweenness is a measure that indicates to what extent a specific node is between all other nodes within the network [44]. In a general sense, betweenness show the influence of a node over the spread of information throughout the network. On the other hand, bottleneck is a local topologic data that is defined as all nodes with high betweenness values and different nodes degrees, indicating that those nodes are central points that control the communication between other nodes within the network [45,46]. The measures of betweenness and node degree allow us to define the bottleneck nodes. Bottleneck nodes correspond to highly central proteins that connect several complexes or are peripheral members of central complexes, being important communication points between two complexes [46]. Mostly of bottleneck nodes tend to be essential proteins in a network [46].

The centrality analysis of union network indicated the presence of 419 bottleneck nodes; 99 of these bottleneck nodes correspond to proteins of genes that were induced in array and 92 that were repressed in array (see Additional file 4: Table A4, spreadsheet 1). The centrality analysis was also made for the 11 clusters (Additional file 4: Table A4, spreadsheet 2). This analysis showed the presence of important bottleneck nodes in the clusters. Several genes that encode many of these proteins characterized as Bottleneck nodes (i. e. essential proteins in a network) were previously described in Tables 2, 3 and Additional file 1: Table A1 once again highlighting the significance of the biological process identified as involved in response and cell tolerance to propolis. Thus, the main bottleneck nodes can be observed in Table 5 and are related to the following biological processes: cellular component organization or biogenesis, transmembrane transport, response to stress (referring to genes induced in the microarray analysis) and chromosome organization, cell cycle, RNA metabolic process (referring to genes repressed in the microarray analysis). To verify the biological processes associated with these genes, see Additional file 1: Table A1.

**Table 5:** Selected bottleneck nodes observed in the union PPPI network

Bottleneck nodes of genes that were found as induced in microarray*	Bottleneck nodes of genes that were found as repressed on microarray*
TIM10	BRN1
RLF2	SMC4
VMA7	RFM1
VMA21	CEP3
ATP17	MAM1
ATP18	NSL1
ATP20	SPC24
AZR1	SLK19
PDR12	ESA1
TPO1	IFH1
TPO4	HOS2
YOR1	LEO1
DFM1	SGF29
GRX4	NGG1
TRX1	IES2
TSA2	
GTT2	
GTT3	
PKR1	
ENA2	
SOP4	

* Indicates genes that were significantly up- or down-regulated in the microarray analysis.

These data express the importance of these pathways in response of *S. cerevisiae* to propolis and confirm a high degree of overlapping in gene function among the microarray hybridization and the system biology analysis. Furthermore, the data obtained here confirms the results achieved through the identification of genes involved in propolis sensitivity by the screening of the *S. cerevisiae* non-essential deletion library previously reported [17].

## Conclusions

Propolis is a complex product derived from plant resins and bee’s saliva. There are several chemical compounds present in this natural product that could potentially be responsible for its antibiotic properties. However, taking into consideration the fact that the cell death effects of propolis could be due to a great combination of chemical compounds and concentrations, we decided to investigate the cell death effects of propolis by concentrating our experiments on alcoholic extracts of propolis In summary, our data indicate that propolis is largely affecting several pathways in the eukaryotic cell. However, the most prominent pathways are related to oxidative stress, mitochondrial electron transport chain, vacuolar acidification, regulation of macroautophagy associated with protein target to vacuole, cellular response to starvation, and negative regulation of transcription from RNA polymerase II promoter. Our work emphasizes again the importance of *S. cerevisiae* as a model system to understand at molecular level the mechanism whereby propolis causes cell death in this organism at the concentration herein tested. Our study is the first one that investigates systematically by using functional genomics how propolis influences and modulates the mRNA abundance of an organism and may stimulate further work on the propolis-mediated cell death mechanisms in fungi.

## Competing interests

The authors declare that they have no competing interests.

## Authors’ contributions

PAC and MS performed most of the experiments. PAC, DB, AB, IM, MHSG and GHG performed data analysis. GHG wrote the manuscript; conceived, designed and coordinated this study; and is the principal investigator of this work. All authors read and approved final manuscript.

## Pre-publication history

The pre-publication history for this paper can be accessed here:

http://www.biomedcentral.com/1472-6882/12/194/prepub

## Supplementary Material

Additional file 1 **Table A1.** Genes with increased or repressed mRNA expression and grouped according to the GO identity. Click here for file

Additional file 2 **Table A2.** Induced- and repressed- genes associated PPPI networks. Click here for file

Additional file 3 **Table A2.** Sub networks present in the union PPPI network and their GO analysis. Click here for file

Additional file 4 **Table A4.** Centrality analysis. Click here for file
